# Linking common non-coding RNAs of human lung cancer and M. tuberculosis

**DOI:** 10.6026/97320630014337

**Published:** 2018-06-30

**Authors:** Debmalya Barh, Sandeep Tiwari, Ranjith N. Kumavath, Preetam Ghosh, Vasco Azevedo

**Affiliations:** 1 Centre for Genomics and Applied Gene Technology, Institute of Integrative Omics and Applied Biotechnology (IIOAB), Nonakuri, Purba Medinipur, West Bengal, India; 2 Laboratorio de Genetica Celular e Molecular, Departamento de Biologia Geral, Instituto de Ciencias Biologicas (ICB), Universidade Federal de Minas Gerais, Pampulha, Belo Horizonte, Minas Gerais, Brazil; 3 Division of Bioinformatics and Computational Genomics, NITTE University Center for Science Education and Research (NUCSER), NITTE (Deemed to be University), Deralakatte, Mangaluru, Karnataka, India; 4 Department of Genomic Science, School of Biological Sciences, Central University of Kerala, Tejaswini Hills, Periya (P.O) Kasaragod, Kerala-671316, India; 5 Department of Computer Science, Virginia Commonwealth University, Virginia 23284, USA

**Keywords:** Genetic predisposition, lung cancer risk, microRNA, Mycobacterium tuberculosis, sRNA

## Abstract

Lung cancer and pulmonary tuberculosis caused by Mycobacterium are two major causes of deaths worldwide. Tuberculosis linked lung cancer is known. However, the precise molecular mechanism of Mycobacterium associated increased risk of lung cancer is not understood. We report 45 common human miRNAs deregulated in both pulmonary tuberculosis and lung cancer. We show that sRNA_1096 and sRNA_1414 from M. tuberculosis have sequence homology with human mir-21. Hence, the potential role of these three small non-coding RNAs in rifampicin resistance in pulmonary tuberculosis is implied. Further, the linking of sRNA_1096 and sRNA_1414 from M. tuberculosis with the host lung tumorigenesis is inferred. Nonetheless, further analysis and validation is required to associate these three non-coding RNAs with Mycobacterium associated increased risk of lung cancer.

## Background

Viral involvements and their causal roles in oncology are well accepted for various cancers including ovarian neoplasms [1] hepatocellular carcinoma [2] and lung cancer [3] among others. Although, bacterial infections are not considered as major threats to cancer, yet a number of bacterial pathogens are reported to be associated with several cancers. Some examples include: Mycoplasma in prostate malignancy [4], Robinsoniella in pancreatic cancer [5], S. typhi, H. bilis, H. hepaticus, and E. coli in carcinoma of the gallbladder [6], Chlamydia in cervical cancer [7] and Mycobacterium in lung cancer [8, 9, 10, 11, 12, 13, 14].

Lung cancer is the leading cause of all cancer related deaths with a recently estimated 1.6 million deaths worldwide [15]. Similar to lung cancer, pulmonary tuberculosis caused by M. tuberculosis is a global health problem. It is one of the major causes of death amongst infectious diseases and according to WHO 2013 report, it is estimated that 9 million people are infected and 1.5 million died from tuberculosis in 2012 [16]. Several reports have documented the co-existence of tuberculosis and lung cancer [14, 17, 18, 19, 20], and pulmonary tuberculosis is a risk factor for developing lung cancer [17, 18, 19, 20]. However, it is not yet fully established at the molecular level, how the Mycobacterium increases susceptibility to lung cancer. Some reports say that M. tuberculosis induces ROS mediated DNA damage pathway and produces epiregulin growth factor to induce cell proliferation [21]; while an other study indicates mechanisms along with COX-2 mediated activation of inflammatory pathway in M. tuberculosis associated carcinogenesis [22] .

Bacterial small regulatory RNAs (sRNAs) are a class of small non-coding RNAs of 40-500 nt in length that regulate various essential patho-physiologies in bacteria such as outer membrane protein biogenesis, virulence, quorum sensing etc. sRNA functions through complementary base-pairing with 3'- or 5'- UTRs of target mRNAs to inhibit translation, alters activity of a protein by directly binding, and by mimicking RNA and DNA structures [23, 24, 25]. Several sRNAs have been identified or predicted from M. tuberculosis [26, 27, 28] having probable role in pulmonary tuberculosis pathogenesis [26, 29, 30]. However, no report so far is available on sRNAs from M. tuberculosis having role in lung cancer. On the other hand, human micro RNAs (miRNAs) are small non-coding RNAs of 20-25 nt length that inhibit post-transcriptional gene regulation by complementary base pairing at the 3' -UTRs of target mRNAs and regulate various patho-physiological conditions including cell cycle regulation, cell differentiation, development, metabolism, aging, different types of cancers, metabolic disorders, and neuronal diseases etc. [31, 32]. Several miRNAs have been implemented to be associated with lung cancer having causative roles and diagnostic potentials [33]. Similarly, a number of miRNAs are found deregulated in pulmonary tuberculosis patients [34, 35, 36].

Since the sRNAs and miRNAs are similar in structure and their mode of actions, and the miRNAs are associated with both pulmonary tuberculosis and lung cancer; we hypothesized that Mycobacterium sRNAs may be associated with lung cancer tumorigenesis. Since Mycobacterium infection is a risk factor in developing lung cancer, here we postulated a "Genetic remittance" hypothesis which presumes that M. tuberculosis sRNAs having similarity with human miRNAs (that are associated with either pulmonary tuberculosis or lung cancer or both diseases) are transmitted to the host during Mycobacterium infection, remained within the human, and act as a predisposition factor to increase the risk of lung cancer.

In this study, using in silico strategies, we aimed to understand (i) the role of M. tuberculosis sRNAs in lung carcinogenesis; (ii) if there are structurally and functionally similar M. tuberculosis sRNAs to human miRNAs responsible for pathogenesis in both pulmonary tuberculosis and lung cancer and their probable functions in these diseases; and (iii) proof of concept of our "Genetic remittance" hypothesis.

## Methodology

### Collection of human miRNAs and M. tuberculosis siRNAs

We used PubMed (http://www.ncbi.nlm.nih.gov/pubmed), miRegulome [32], miR2Disease [37], TUMIR [38], sRNAdb [39], and BSRD [40] databases to get the data. The data deposited in these databases during January 2006 to November 2015 were searched. In the first step, we collected all the validated deregulated miRNAs associated with lung cancer and pulmonary tuberculosis from published literature indexed in PubMed (http://www.ncbi.nlm.nih.gov/pubmed) and from databases such as miRegulome [32], miR2Disease [37], and TUMIR [38]. The common miRNAs associated with both the diseases were manually identified and listed out separately. Similar to miRNA, we collected all reported and novel M. tuberculosis sRNAs by means of PubMed literature mining and searching sRNA databases such as sRNAdb [39] and BSRD [40]. For PubMed search, specific key words such as "miRNA + M. tuberculosis", "miRNA + tuberculosis", "miRNA + lung cncer", sRNA + M. tuberculosis", "sRNA + tuberculosis" etc. were used.

### Prediction of miRNAs that may function as sRNA or vice versa

To achieve this, we used a simple strategy. Since the sRNAs and miRNAs are very short in sequence, we presumed that small sequence motifs in these non-coding RNAs are very important for their specific functions. Therefore, we used comparative BLASTn (miRNA against sRNA) with default parameters in order to identify if there is any human miRNA (from the group of miRNAs common to both lung cancer and pulmonary tuberculosis) having sequence similarity to any M. tuberculosis sRNA, so they may have similar functions. From the BLASTn results, the specific motif sequences that are common in sRNAs and miRNAs were considered.

### Functional annotation of M. tuberculosis sRNAs

The functional annotation of the sRNAs was carried out using target based reverse annotation approaches following a modified protocol as described by Barh et al., 2013 [33]. In brief, we identified the validated targets of sRNA using sRNATarBase [41] database and RNApredator [42] was used to predict putative targets in M. tuberculosis, H37Rv. Top 100 targets were used for functional annotation by using the DAVID functional annotation tool [43]. Further, we presumed that, if there is a coding gene that has identity with sRNA; the function of the sRNA could be similar to that gene. Therefore, we performed sRNA BLASTn against M. tuberculosis genome using NCBI BLASTn server (http://blast.ncbi.nlm.nih.gov/Blast.cgi) to identify if there is any coding sequence present in M. tuberculosis genome having sequence identity similar to that of sRNA. To check if the sRNA is targeting an essential gene of M. tuberculosis, H37Rv, we used Database of Essential Genes (DEG) [44] BLASTn and to check if the target could be a drug target, we used the strategy as described in [45]. Further, we did miRNA (that is having sequence similarity with sRNA) BLASTn against M. tuberculosis genome in NCBI BLASTn server with default parameters to check if any M. tuberculosis coding sequence is matching with the miRNA sequence. Since, identified miRNA in this way share sRNA sequences if there is a coding sequence that matches with the miRNA, we consider that, the matched coding sequence of the sRNA and the miRNA may have similar function.

### Genetic remittance

We performed BLASTn of the M. tuberculosis sRNAs having sequence similarity with human miRNA (that are associated with both pulmonary tuberculosis and lung cancer) against the human genome on NCBI BLASTn server in order to identify if there is any human coding sequence having identity with the sRNAs. Thus, common miRNAs that are deregulated both in pulmonary tuberculosis and lung cancer is observed. These observation indicate genetic remittance in tuberculosis associated lung cancer.

## Results

### Common miRNAs in pulmonary tuberculosis and lung cancer

From various literature and databases, we collected differential expression of 186 human miRNAs in pulmonary tuberculosis and 242 miRNAs in lung cancer patients. However, while we checked for the common miRNAs that are deregulated both in pulmonary tuberculosis and lung cancer, we found the number is only 45 Table 1 (see supplementary data).

### M. tuberculosis sRNA_1096 and sRNA_1414 shares hsa-mir-21 sequence

From the sRNAdb [39] and BSRD [40] databases and literature [27, 46] we collected 120 reported M. tuberculosis sRNAs and their sequences. The comparative BLASTn between the 120 sRNAs and the common 45 human miRNAs in pulmonary tuberculosis and lung cancer revealed that the mature human miRNA hsa-mir-21- 5p has very short sequence similarity with M. tuberculosis sRNA_1096 and sRNA_1414. Both these sRNAs are experimentally validated in M. tuberculosis [27]. Human hsa-mir- 21-5p and M. tuberculosis sRNA_1096 share a common sequence of GTTG/ GUUG, while the common sequence between hsa-mir- 21-5p and M. tuberculosis sRNA_1414 is ATCAG/ AUCAG Table 2 (see supplementary data).

### Functional annotation of sRNA_1096 and sRNA_1414:

To understand the functions of sRNA_1096 and sRNA_1414 in M. tuberculosis, first we used sRNATarBase [41] to search validated targets of these sRNAs. However we did not get any target from this database. Therefore, we used RNApredator [42] to predict the targets of these sRNAs. The top 100 targets based on the Zscore of RNApredator were further used for functional annotation using DAVID.

For sRNA_1096, phosphate-binding protein pstS 2 (Z-score: - 11.29) was found to be the best target. pstS 2 is an inorganic phosphate transmembrane transporter that is involved in twocomponent system and ABC transporters pathways. Further, among the top 100 targets, fourteen targets are PE PGRS family proteins Table 3 (see supplementary data). DAVID functional annotation analysis of the top 100 targets of sRNA_1096 shows that most targets are membrane located and the two-component system is the top annotation cluster.

The top target of sRNA_1414 is found to be glycyl-tRNA synthetase / glycine--tRNA ligase (glyS) (Z-score: -11.41) by RNApredator [42] (Table 3, available with author). The drug transporter activity is ranked as the first annotation cluster and most targets are transmembrane proteins as observed through DAVID functional annotation analysis. According to the Database of Essential Genes (DEG) [44], the M. tuberculosis glyS is an essential gene in the pathogen but has 31% identity at protein level with human glycine--tRNA ligase as per NCBI human BLASTp.

When we performed sRNA_1096 BLASTn against M. tuberculosis genome, a very short similarity "CCGTCACCGTTG" was observed with M. tuberculosis Arabinosyltransferase EmbC (embC) gene. The BLASTn of sRNA_1414 with M. tuberculosis genome did not show any hit with any specific protein-coding gene of the pathogen.

### Common function of sRNA_1096, sRNA_1414, and hsa-mir-21:

As in previous analysis we found there are sequence similarities among hsa-mir-21 and M. tuberculosis sRNA_1096 and sRNA_1414; we performed a NCBI BLASTn of hsa-mir-21-5p against the M. tuberculosis genome. We observed that the M. tuberculosis rpoB gene that provides rifampin/ rifampicin resistance in M. tuberculosis has sequence similarity with mir-21 and has both the GTTG and ATCAG short-stretch sequences that are present in M. tuberculosis sRNA_1096 and sRNA_1414, respectively. Therefore, all these non-coding RNAs may be involved in rifampicin resistance in pulmonary tuberculosis.

### Support for "Genetic remittance" hypothesis

To support our "Genetic remittance" hypothesis, we tried to identify if there is any sequence match of sRNA_1096 and sRNA_1414 in human coding sequence. The BLASTn of sRNA_1096 against human genome shows a hit with SH3GL1 (SH3-domain GRB2-like 1) and some sequence of sRNA_1414 matches with human EPS8L1 (EPS8-like 1) and SORBS1 (Sorbin and SH3 domain containing 1). All these human genes are associated with tumorigenesis, thus providing a preliminary support to our hypothesis.

## Discussion

In this study we found that there could be correlations between lung cancer and pulmonary tuberculosis at the non-coding RNA level. M. tuberculosis sRNA_1096 and sRNA_1414, and human hsa-mir-21-5p are probably the links to explain why the pulmonary tuberculosis is a risk factor in developing lung cancer. Among the 45 human miRNAs that are deregulated both in both the diseases Table 1 (see supplementary data) and 120 reported M. tuberculosis sRNAs in the M. tuberculosisis genome, we found that there are sequence similarities among human hsa-mir-21-5p and M. tuberculosis sRNA_1096 and sRNA_1414 Table 2 (see supplementary data).

The oncomiR hsa-mir-21 is frequently upregulated in lung cancer [47, 48, 49, 50, 51, 52, 53, 54, 55] while it is downregulated in CD4+ T cells in tuberculosis patients [56]. However, hsa-mir-21 is found upregulated in the host during M. bovis BCG infection [57] Table 1 (see supplementary data). It is also observed that mir-21 plays a role in T-cell immunity against M. tuberculosisis [56] and found to affect the anti-mycobacterial T- cell response through targeting IL12 and BCL2 [57]. Reports suggest that the bacteria induced carcinogenesis occurred in multiple ways. These mechanisms include induction or interference of chronic inflammatory and other signalling cascades including TLRs (Toll-like receptors) signalling and acetaldehyde metabolism pathways in various cancers [58, 59, 60]. TLRs signalling are generally involved in innate and adaptive immune responses. However, activation of TLRs signalling promotes tumor cell proliferation, growth, invasion, and metastasis [61]. In lung cancer, activation of TLR7 and TLR8 increases survival and chemoresistance of the tumor cells; therefore these two TLRs could be targets for tumor immunotherapy [62]. In Mycobacterial infection, TLRs signalling regulates host innate and inflammatory responses and determines the disease outcome [63]. Polymorphisms and over expression of TLR8 is associated with pulmonary tuberculosis susceptibility and infection, respectively [64]. However, the precise mechanism of TLR8 in pulmonary tuberculosis is not yet known [65]. Fabbri et al in 2012 first reported the novel mechanism of oncomiR mir-21 that can acts as a ligand for TLR8 to induce inflammatory response leading to tumor growth and metastasis [66]. This study also showed that the “GUUG” motif miR-21 directly binds to TLR8 to induce the TLR-mediated prometastatic inflammatory response.

### M. tuberculosis sRNA_1096

In our analysis, we found that the GTTG/ GUUG sequence is conserved in hsa-miR-21 and experimentally validated M. tuberculosis sRNA_1096 (Table 2 (see supplementary data)). Therefore, it may be implicated that the M. tuberculosis sRNA_1096 may bind to TLR8 in a similar way as miR-21 does through its GUUG motif and thus may activate immune and inflammatory responses and explains a role of TLR8 in pulmonary tuberculosis.

Further, as per our analysis, M. tuberculosis sRNA_1096 may play a role in two-component system pathway and may regulate PE PGRS family and membrane located proteins. PE PGRS family proteins act as variable surface antigens and are involved in multiple levels of the infectious process, modulation of innate immune responses [67], and virulence [68] in M. tuberculosis. Similarly, the two-component system is crucial in Mycobacterial survival and pathogenicity [69]. In M. tuberculosis genome, "CCGTCACCGTTG" sequence of the sRNA_1096 is present in Arabino-syl transferase EmbC (embC) gene, which is involved in bacteria-host interactions and also modulates immune response in M. tuberculosis [70]. Therefore, M. tuberculosis sRNA_1096 could play an important role in M. tuberculosis pathogenesis leading to pulmonary tuberculosis. In support to our "Genetic remittance" hypothesis, we predicted that if the M. tuberculosis sRNA_1096 is present or predisposes to human, it may modulate human SH3GL1 (SH3-domain GRB2-like 1) which plays a role in endocytosis [71], regulation of cell cycle in leukaemia [72], positive regulation of cell proliferation and inhibition of apoptosis in multiple myeloma [73], and oncogenesis in gliomas [74]. Hence, sRNA_1096 may play a critical role in lung cancer risk. Further, if the sRNA_1096 acts as a ligand to TLR8 similar to mir-21, upon remittance to the host, it may activate TLR8 pathway and along with increasing risk it may also regulate chemoresistance [62] in lung cancer individually or in combination with mir-21. Therefore, we postulate that, the M. tuberculosis sRNA_1096 is involved in pulmonary tuberculosis pathogenesis through multiple infectious processes including TLR8 mediated pathway. The sRNA_1096 may be transported to host and predisposed during M. tuberculosis infection and later acts as ligand to TLR8 through its GTTG/ GUUG sequence similar to mir-21 to activate TLR8 mediated prometastatic pathways and chemoresistance in lung cancer. Similar to SH3GL1, it may also regulate tumorigenic inflammatory response and cell cycle, respectively leading to lung cancer. Hence, sRNA_1096 may be an emerging marker for tuberculosis and lung cancer risk and chemoresistance in combination with mir-21.

### M. tuberculosis sRNA_1414

On the other hand, we observed ATCAG/ AUCAG as the common sequence between hsa-mir-21-5p and M. tuberculosis sRNA_1414 Table 2 (see supplementary data). The sRNA_1414 is predicted to target glycyl-tRNA synthetase (glyS), which is an essential gene in M. tuberculosis. Further, we observed that sRNA_1414 might regulate drug transporter activity. Aspartyl-tRNA synthetase and tyrosyl-tRNA synthetase are important drug targets in M. tuberculosis [75, 76] and polymorphisms in aspartyl-tRNA synthetase is associated with drug resistance mechanism in this pathogen [77]. Although, we predicted glyS is as an essential gene in M. tuberculosis, being a human homolog, it is not a suitable target. Thus sRNA_1414 may probably be involved in regulating the survivability and drug response of the pathogen.

We found sequence matches with human EPS8L1 (EPS8-like 1) and SORBS1 (Sorbin and SH3 domain containing 1) by BLASTn of sRNA_1414 against human genome. This indicates genetic remittance in tuberculosis associated lung cancer. EPS8L1 encodes a protein that is related to epidermal growth factor receptor pathway substrate 8 and is involved in regulation of Rho protein signal transduction [78], which is associated with small cell lung cancer migration [79]. Similarly, SORBS1 (Sorbin and SH3 domain containing 1) plays an important role in cell-matrix adhesion [80], a key process in cell migration. Therefore, if the M. tuberculosis sRNA_1414 is transferred to the host during the infection, it may lead to lung cancer metastasis in later stage functioning similar to EPS8L1 and SORBS1.

### Common functions of M. tuberculosis sRNA_1096 and sRNA_1414

Since the hsa-mir-21 sequence GTTG /GUUA is shared by sRNA_1096 and ATCAG/ AUCAG by sRNA_1414 Table 2 (see supplementary data), we tried to predict the common role of these non-coding RNAs. Our hsa-mir- 21 BLASTn against M. tuberculosis shows that these GTTG /GUUA and ATCAG/ AUCAG sequences are present in M. tuberculosis rpoB gene that provides rifampin/ rifampicin resistance in M. tuberculosis [81, 82, 83, 84]. Therefore, we presume that all these non-coding RNAs: hsa-mir-21, sRNA_1096, and sRNA_1414 could be involved in rifampicin resistance and an up regulation of mir-21 in tuberculosis patient may be a marker of rifampicin resistance.

## Conclusion

**Figure 1:**
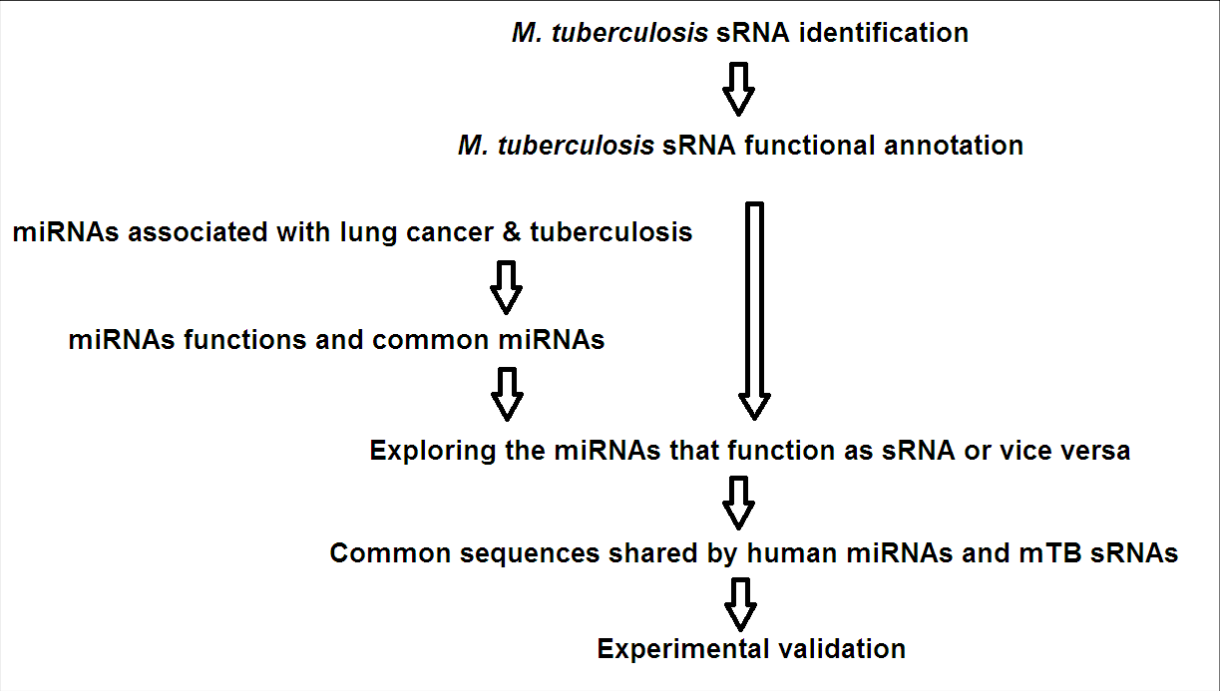
The integrated bioinformatics and experimental validation strategy to elucidate the relationship between tuberculosis and lung cancer linked through non-coding RNAs.

M. tuberculosis sRNA_1096 involvement in tuberculosis through multiple molecular processes is of interest to know. This is through the potential activation of TLR8 mediated pro-metastatic inflammatory pathway by acting as a ligand to TLR8. This is similar to mir-21 action leading to lung tumorigenesis and subsequent chemo-resistance. The role of sRNA in cell cycle regulation similar to the human SH3GL1 is relevant. The role of sRNA_1414 in survivability and drug response of the pathogen is contextual. The three non-coding RNAs are predicted to act in rifampicin resistance against Mycobacterium. Further data analysis as outlined in Figure 1 including domain/ motif analysis along with experimental validations are required to validate the observation.

## Funding

The authors have no support or funding to report

## Competing interests

The authors have no competing interests.

## Author contributions

Conceived, designed the experiment, collected and analyzed initial data, coordinated the entire project: DB, Performed all analysis: DB, ST, PG, RK, Wrote the paper: DB. VA guided the project. All authors read and approved the manuscript.

## Supplementary material

Data 1
