# Supplementary material for: Linking common non-coding RNAs of human lung cancer and M. tuberculosis
Source: Bioinformation. 2018 Jun 30;14(6):337–45. doi: 10.6026/97320630014337 (PMC6137563; doi:10.6026/97320630014337)
Supplement: Data 1 [file 97320630014337S1.pdf]

BIOINFORMATION
Discovery at the interface of physical and biological sciences

Open access

Supplementary Data:

Supplementary Table S1:
Common miRNAs that are deregulated both in pulmonary tuberculosis and lung cancer.

miRNA Name	Regulation in lung cancer	Reference (PMID)/doi	Regulation in Tuberculosis	References (PMID)/doi
hsa-let-7g	Down-regulated	doi: 10.4046/trd.2009.67.5.413, 16461460; 18766170	Up-regulated (serum)	21998423
hsa-let-7i	Down-regulated	18766170	Up-regulated	21998423
hsa-miR-21	Up-regulated	19228723, 19273703, 18766170, 18719201, 21116241, 19493678, 16530703, 16461460, 21351266	Down-regulated<br>Up-regulated in Mycobacterium bovis BCG infection	23613882, 22710123
hsa-miR-23b	Down-regulated	2907339	Up-regulated	21998423
hsa-miR-23b	Up-regulated	18766170	Deregulated	
hsa-miR-22	Down-regulated	2907339; 18766170	Up-regulated	21998423
hsa-miR-26a	Up-regulated	18766170	Down-regulated	23613882
hsa-miR-26a			Up-regulated	22087245
hsa-miR-26b	Down-regulated	18766170	Deregulated	doi:10.4236/jtr.2013.12 005
hsa-miR-29a	Down-regulated	2907339; 17890317	Down-regulated	23613882
hsa-miR-29a			Up-regulated	23613882; 21998423; 21785411
hsa-miR-30c	Down-regulated	doi: 10.4046/trd.2009.67.5.413	Up-regulated	21998423
hsa-miR-99b	Up-regulated	16461460; doi: 10.4046/trd.2009.67.5.413	Up-regulated	23233675
hsa-miR-101	Up-regulated	2907339	Up-regulated	21998423
hsa-miR-101	Down-regulated	18766170, doi: 10.4046/trd.2009.67.5.413		
hsa-miR-101	Down-regulated	16530703		
hsa-miR-103	Up-regulated	2907339; 18766170	Up-regulated	21998423
hsa-miR-125a-5p	Down-regulated	20569443	Up-regulated	23233675
hsa-miR-125a-5p	Down-regulated	20569443, 18766170		
hsa-miR-125a-5p	Down-regulated	16530703; 19584273, doi: 10.4046/trd.2009.67.5.413		
hsa-miR-125b	Up-regulated	18766170, doi: 10.4046/trd.2009.67.5.413	Up-regulated	23448104; 21998423; 17911593
hsa-miR-133a	Down-regulated	doi: 10.4046/trd.2009.67.5.413	Up-regulated	22003408
hsa-miR-134	Up-regulated	18766170	Up-regulated	23272999; 22087245

hsa-miR-142-3p	Up-regulated	2907339	Down-regulated	23613882
hsa-miR-142-3p	Down-regulated	19228723, doi: 10.4046/trd.2009.67.5.413		
hsa-miR-143	Down-regulated	19895320, 16530703	Up-regulated	21998423
hsa-miR-144	Down-regulated	doi: 10.4046/trd.2009.67.5.413	Up-regulated	22003408
hsa-miR-146a	Up-regulated	18766170	Up-regulated	21998423
hsa-miR-148b	Up-regulated	2907339	Down-regulated	24084739
hsa-miR-155	Down-regulated	19895320	Up-regulated	21367459; 22712528; 24130493
hsa-miR-155	Up-regulated	16530703, 16461460	Down-regulated	22003408; 21969554
hsa-miR-181b	Up-regulated	18766170	Down-regulated	21998423; 22003408
hsa-miR-191	Up-regulated	18766170, 16461460, 16530703	Up-regulated	21998423
hsa-miR-197	Up-regulated	18766170; 16530703	Up-regulated	24084739
hsa-miR-206	Up-regulated	18766170	Down-regulated	21998423
hsa-miR-210	Up-regulated	20526284, 21116241, 19493678, doi: 10.4046/trd.2009.67.5.413, 16461460, 16530703	Up-regulated	23272999; 22087245
hsa-miR-212	Up-regulated	16530703	Deregulated	doi:10.4236/jtr.2013.12 005
hsa-miR-218	Down-regulated	20838434	Up-regulated	21998423
hsa-miR-222	Down-regulated	2907339, 19895320	Up-regulated	23233675
hsa-miR-222	Up-regulated	19962668, 18766170		
hsa-miR-223	Down-regulated	19895320	Up-regulated	24084739; 22003408
hsa-miR-223	Up-regulated	18766170		
hsa-miR-296-5p	Down-regulated	doi: 10.4046/trd.2009.67.5.413	Down-regulated	24084739
hsa-miR-371-3p	Up-regulated	doi: 10.4046/trd.2009.67.5.413	Down-regulated	21998423
hsa-miR-375	Up-regulated	18766170, 21351266	Up-regulated	21998423
hsa-miR-382	Up-regulated	18766170	Up-regulated	21998423
hsa-miR-423-5p	Upregulated	18766170	Up-regulated	22087245
hsa-miR-432	upregulated	18766170	Up-regulated	23272999; 22087245
hsa-miR-433	Up-regulated	18766170	Up-regulated	21998423
hsa-miR-451	Down-regulated	18766170	Down-regulated	24084739; 22003408
hsa-miR-483-5p	Up-regulated	18766170	Up-regulated	21998423
hsa-miR-486-5p	Down-regulated	21116241, 20194856	Up-regulated	22003408
hsa-miR-501-3p	Up-regulated	18766170	Down-regulated	24084739

BIOINFORMATION
Discovery at the interface of physical and biological sciences

Open access

hsa-miR-574-5p	Up-regulated	21258252	Up-regulated	21998423
hsa-miR-629	Up-regulated	18766170	Up-regulated	24084739
hsa-miR-744	Up-regulated	18766170	Down-regulated	24084739
hsa-miR-744			Up-regulated	21998423

Supplementary Table S2:
Sequences and similarities among of *M. tuberculosis* sRNA_1096, sRNA_1414, and human hsa-miR-21

M. tuberculosis sRNA_1096
CGAGCCGTCACC**GTTG**TGCATCGAAAGAGGTCTGATC

M. tuberculosis sRNA_1414
GGCAGACGCGCGCAGCCCGACACGACTACGCGCAAAACATCAGTCA

hsa-miR-21-5p
TAGCTTATCAGACTGAT**GTTG**A

The GUUG / GTTG sequence of mir-21 is involved in binding with TLR8 (PMID: 22753494)

hsa-miR-21-5p
UAGCUUAUCAGACUGAU**GUUG**A

Supplementary Table S3: (Available with author)
RNApredator based predicted targets of *M. tuberculosis* sRNA_1096 and sRNA_1414.
